# Genotype diversity and molecular evolution of noroviruses: A 30-year (1982-2011) comprehensive study with children from Northern Brazil

**DOI:** 10.1371/journal.pone.0178909

**Published:** 2017-06-12

**Authors:** Jones Anderson Monteiro Siqueira, Renato da Silva Bandeira, Darleise de Souza Oliveira, Liann Filiphe Pereira dos Santos, Yvone Benchimol Gabbay

**Affiliations:** 1 Laboratório de Norovírus e outros Vírus Gastroentéricos—LNVE, Seção de Virologia—SAVIR, Instituto Evandro Chagas—IEC, Secretaria de Vigilância em Saúde, Ministério da Saúde, Ananindeua, Pará, Brazil; 2 Seção de Virologia–SAVIR, Instituto Evandro Chagas—IEC, Secretaria de Vigilância em Saúde, Ministério da Saúde, Ananindeua, Pará, Brazil; Centers for Disease Control and Prevention, UNITED STATES

## Abstract

A chronologically comprehensive 30-year study was conducted that involved children living in Belém, in the Amazon region of Northern Brazil, who participated in eight different studies from October 1982 to April 2011. The children were followed either in the community or in health units and hospitals in order to identify the norovirus genotypes involved in infections during this time. A total of 2,520 fecal specimens were obtained and subjected to RT-PCR and nucleotide sequencing for regions A, B, C, D and P2 of the viral genome. An overall positivity of 16.9% (n = 426) was observed, and 49% of the positive samples were genotyped (208/426), evidencing the presence of several genotypes as follows: Polymerase gene (GI.P4, GII.Pa, GII.Pc, GII.Pe, GII.Pg, GII.Pj, GII.P3, GII.P4, GII.P6, GII.P7, GII.P8, GII.P12, GII.P13, GII.P14, GII.P21, GII.P22), and VP1 gene (GI.3, GI.7, GII.1, GII.2, GII.3, GII.4, GII.6, GII.7, GII.8, GII.10, GII.12, GII.14, GII.17, GII.23). The GII.P4/GII.4 genotype determined by both open reading frames (ORFs) (partial polymerase and VP1 genes) was found for 83 samples, and analyses of the subdomain P2 region showed 10 different variants: CHDC (1970s), Tokyo (1980s), Bristol_1993, US_95/96, Kaiso_2003, Asia_2003, Hunter_2004, Yerseke_2006a, Den Haag_2006b (subcluster “O”) and New Orleans_2009. Recombination events were confirmed in 47.6% (n = 20) of the 42 samples with divergent genotyping by ORF1 and ORF2 and with probable different breakpoints within the viral genome. The evolutionary analyses estimated a rate of evolution of 1.02 x 10^−2^ and 9.05 x 10^−3^ subs./site/year using regions C and D from the VP1 gene, respectively. The present research shows the broad genetic diversity of the norovirus that infected children for 30 years in Belém. These findings contribute to our understanding of noroviruses molecular epidemiology and viral evolution and provide a baseline for vaccine design.

## Introduction

Historically, noroviruses (NoVs) were first detected in samples derived from an acute gastroenteritis (AGE) outbreak at an elementary school in Norwalk, OH, USA in 1968 [1]. Despite a long history of study, knowledge about the epidemiological dynamics responsible for the constant generation of genetic and antigenic heterogeneity that allows NoVs to evade the host immune response and which might be responsible for their persistence in the human population needs to be better understood [2].

NoVs are well recognized as primarily responsible for AGE outbreaks in isolated settings (e.g., nursing homes, hospitals, day care centers, cruise ships, restaurants, etc.) [3], especially in health care institutions, where the most serious cases that resulted in deaths, especially of the elderly (80 deaths per year in people more than 64 years of age) have occurred [4]. However, other high risk groups, such as children, travelers, soldiers and immunocompromised patients, also exist [5,6].

It is estimated that each year in the pediatric population, approximately 200,000 deaths of children less than 5 years of age occur in economically developing countries; however, the epidemiological relevance of NoVs in populations of low income countries should be more rigorously investigated [6]. In a systematic review that assessed original data from 31 countries (11 of which are currently undergoing socio-economic development) between January 1990 and February 2008, it was estimated that 12% of all cases of sporadic AGE in children were associated with NoVs [7].

The use of molecular diagnostics worldwide and the number of studies that have evaluated the role of NoVs in episodes of sporadic AGE in several countries, including those with high or low per capita income, have recently increased substantially, allowing an update of such data [6]. A meta-analysis of 175 articles involving over 187,000 patients showed that 18% (95% confidence interval [CI] = 17–20) of the overall NoVs-prevalence related to AGE was higher at the community level (24% [95% CI = 18–30]) and for outpatients (20% [95% CI = 16–24]) than for hospital patients [17% (95% CI = 15–19)] [8].

The norovirus (NoV) has a nonenveloped icosahedral capsid of approximately 27 to 40 nm in diameter that surrounds a positive-sense single-stranded RNA genome with a genome length of 7.3 to 7.5 kb, which is organized into three open reading frames (ORFs) (i.e., ORF1, ORF2 and ORF3) [9] that include all regions (A to E) that have been used for genotyping by different research groups worldwide [10].

Genetically, the genus *Norovirus* is one of five genera that belong to the family *Caliciviridae*, and it is divided into seven genogroups (GI-GVII), of which three (GI, GII and GIV) have been associated with AGE in humans. These genogroups are composed of 41 genotypes [11], and GII.4 is the most prevalent worldwide and has been associated with the majority of global outbreaks since the mid-1990s, when active surveillance using molecular diagnostic techniques was initiated [12,13].

Studies that aim to define the genotypic variability of NoV are important to establish a more complete epidemiological-molecular picture. Such studies will allow an understanding of infection dynamics, particularly with regard to the detection of variant strains, especially those related to genotype GII.4, which has been the target of the primary studies aimed at the production of NoV vaccines. In addition, knowledge of the circulating recombinant genotypes is fundamental for a better understanding of the evolutionary pattern of this pathogen, since this genetic phenomenon has been considered the main driving force of NoV evolution, which has often been associated with the emergence of new strains circulating in populations worldwide.

In this context, we conducted a chronologically comprehensive 30-year study that involved children who we followed either in the community as attended outpatients or in hospitals in Belém, in the Amazon region of Northern Brazil, to identify the primary NoV genotypes involved in AGE cases and in some cases of infection in the controls. We felt that by understanding the diversity of strains found over such a long period, we might gain insight into the likelihood that vaccines being developed today could adequately address the plethora of strains identified from 1982 to 2011. We also hoped to uncover which evolutionary mechanisms occurred in this collection of long-term samples, which was the primary goal of this retrospective approach about the molecular epidemiology and evolution of NoV.

## Material and methods

### Study design

Fecal samples were collected from children who participated in eight different studies (three cross-sectional studies, three randomized clinical trials, one case-control study and one longitudinal study) conducted by the Evandro Chagas Institute in Belém, Pará, Amazon region, Northern Brazil, from 1982 to 2011 that involved hospitalized or outpatient children and infants in the community. A total of 10,238 samples were collected, and a subset of 2,396 fecal specimens was selected from children who presented clinical symptoms of AGE; 124 control samples were also obtained from children in the community. Selection of the control subjects is more detailed in Siqueira et al. [14]. A total of 2,520 stool samples were enrolled; all had been previously tested for rotavirus (RV), and some had been tested for astrovirus (HAstV). AGE cases were characterized as liquid or semi-liquid stools and three or more bowel movements occurring in a 24-hour period. A summary of the information about each of the studies considered in this investigation is presented in Table 1.

**Table 1:** Summary information for eight studies conducted from 1982 to 2011, the fecal samples of which were included in the present study.

	Study	Period	Description of samples origin	References^*^	GenBank acession number^**^
A	Longitudinal	1982–1986	A community-based longitudinal study that followed children from birth to three years old to investigate the epidemiology and clinical aspects of the rotavirus A (RVA) infection. Samples were previously tested for norovirus (NoV) (16.1%, 37/229).	Siqueira et al. [14] Linhares et al. [15]	KX702004—KX702021 KX452701—KX452716
B	RRV-TV	1990–1992	A randomized clinical trial to evaluate the immunogenicity, safety and efficacy of three doses of quadrivalent reassortant RVA vaccine of simian-human origin (Rotashield, Wyeth Laboratories, Marietta, Pennsylvania).	Linhares et al. [16]	KX702045—KX702046 KX722244—KX722245 KX722392 to KX722399 KX702126
C	Nosocomial	1992–1994	A cross-sectional study conducted at a pediatric hospital to determine the role of different subgroups, serotypes and electropherotypes of RVA in nosocomial cases of acute gastroenteritis (AGE).	Gusmão et al. [17]	KX722400 to KX722406 KX702121
D	Sentinel	1998–2000	A cross-sectional study conducted at a hospital and in a health unit to determine the antigenic diversity of RVA and its current epidemiological impact in Belém, Brazil.	Soares et al. [18]	KX702047 to KX702081 KX722246 to KX722270 KX722321 to KX722341 KX722407 to KX722413 KX702120
E	RIX 4414	2001–2002	A randomized clinical trial to evaluate the immunogenicity, safety and efficacy of two doses of an attenuated monovalent RVA vaccine of human origin (strain RIX 4414).	Araújo et al. [19]	KX702123 to KX702125 KX722271 to KX722274 KX722342 to KX722349
F	Epidemiological surveillance	2003	A cross-sectional study conducted at several emergency departments of health facilities in Belém, Brazil during a prospective multicenter study that aimed to estimate the epidemiology and impact of severe AGE infections caused by RVA in Latin America. The samples were previously tested for NoV (9.8%, 30/305).	Abate et al. [20] Aragão et al. [21]	KX722275 to KX722294 KX722350 to KX722361 KX722414 to KX722424 KX702122
G	Vaccine-Phase III	2004–2005	A randomized clinical trial to ensure the efficacy and safety of a vaccine developed in a multicenter study with healthy children who received two doses of oral attenuated vaccine against RVA at 2 and 4 months of age.	Ruiz-Palacios et al. [22]	KX702092 to KX702112 KX702127 to KX702129 KX722295 to KX722301 KX722362 to KX722367 KX722425 to KX722436
H	Effectiveness	2008–2011	A case-control study conducted at a reference pediatric clinic to evaluate the overall and partial effectiveness of two doses of an RV1 vaccine (Rotarix^®^, GlaxoSmithKline, Rixensart, Belgium), which has been commercially available since March 2006, in the Brazilian National Vaccination Schedule.	Justino et al. [23] Siqueira et al. [24]	KX702022 to KX702044 KX702083 to KX702091 KX702113 to KX702119 KX722302 to KX722320 KX722368 to KX722391 KX722437 to KX722451

*Previously published article that used these same samples and reported rotavirus results, although some reported norovirus results.

**Norovirus sequences obtained in the present study.

### Ethics statement

In the most of studies included in this research, the consent of parents or guardians of the minors were obtained by written before the collection of the samples. In those studies in which the collection samples were made before 1996, the consent was waived by the ethics committee considering the resolution CNS 466/2012 that regulates the use of consents in Brazilian researches with samples collected before that time. This study was approved by the Ethics Review Committee (CEP) of the Evandro Chagas Institute (IEC), under number CAAE: 11988512.8.0000.0019 (OPN. 216.137) dated March 3^rd^, 2013.

### Laboratorial procedures

#### Screening tests

NoV was analyzed by three different methods. Samples from study A were tested by real time RT-PCR (qRT-PCR) as described by Siqueira et al. [14]. Samples from studies B, E, G and H were screened for the presence of NoV antigens using a third-generation commercial Ridascreen Norovirus enzyme immunoassay (EIA) (R-Biopharm, Darmstadt, Germany) according to the manufacturer's instructions, and positive samples were subsequently tested by RT-PCR. Studies C, D and F were initially tested by RT-PCR as described herein.

#### RNA extraction

Viral RNA was extracted using the isothiocyanate guanidine (silica) method [25] or a commercial kit, i.e., the QIAamp Viral RNA Mini Kit (QIAGEN, Germantown, MD, USA) or the PureLink Viral RNA/DNA Mini Kit (Invitrogen, Carlsbad, CA, USA), according to the manufacturer's instructions.

#### cDNA synthesis

Random hexamers [9 A_260_ units/µl, 3 mM Tris-HCl (pH 7.0), and 0.2 mM EDTA—Invitrogen, Eugene, OR, USA] were used in the reverse transcription reaction to obtain complementary DNA (cDNA). If a low yield was obtained in the cDNA synthesis, we used a One-step RT-PCR SuperScript III—Platinum Taq kit (Invitrogen, Carlsbad, CA, USA), in which a single synthesis step is performed and is followed by DNA amplification. This method is thought to be a more stable reaction that produces amplicons of better quality.

#### PCR amplification and genotyping

PCR amplifications were conducted to detect and genotype NoV using primers for regions A (P289/P290, 319 bp; JV13I/JV12Y, 327 bp; JV13I/G1, 187 bp; JV12Y/NoroII-R, 236 bp) or B (431/432/433/434, 213 bp) from the polymerase gene or C (COG2F/G2-SKR, 390 bp) or D (Cap C/D1/D3, 253 bp; Cap A/B1/B2, 177 pb) from the VP1-capsid gene. GII.4 positive samples for both regions (polymerase and VP1) were further analyzed by the amplification of the P2 region (EVP2F-P2/EVP2R-P2, 673 bp) to assess the circulating variants [26–34].

All successfully amplified samples were purified using a QIAquick PCR or Gel extraction purification kit (QIAGEN, Germantown, MD, USA). Cycle sequencing was performed on an ABI Prism 3130XL DNA Sequencer (Applied Biosystems, Foster City, CA, USA) with a Big Dye Terminator kit (v. 3.1; Applied Biosystems, Foster City, CA, USA). In this study, we followed the latest NoV nomenclature for the strain genotypes [35].

### Bioinformatic procedures

#### Phylogenetic analysis and accession numbers

The sequences obtained were aligned using MAFFT (v. 7) [36] and edited in Aliview [37]. Maximum likelihood/neighbor-joining methods and a bootstrap test with 1,000 replicates were applied to support the analysis. The model tests were chosen by IQ-Tree (v. 1.0.1) software [38], and dendrograms were edited and constructed with the FigTree (v. 1.4.2) program [39]. The sequences characterized in this study were submitted to the GenBank database (National Center for Biotechnology Information, USA-[www.ncbi.nlm.nih.gov]) and are provided in Table 1.

#### Recombination analyses

When a difference between polymerase and VP1 genotyping was verified for the same sample, the junction region fragment (431/432/G2-SKR, 544 bp) corresponding to the overlap of ORF1 and ORF2 was analyzed to identify the breakpoint from possible recombination events [26,27,31]. These genetic events were confirmed by an analysis using the SimPlot software (v. 3.5.1) [40] and by RDP/Bootscan/MaxChi methods [41,42] using the Recombination Detection Program (RDP4) Beta (v. 4.6) [43].

#### Evolutionary analyses

These analyses were performed to determine the rate of nucleotide substitutions per site per year (subs./site/year) in those samples genotyped by regions C and D from the VP1 gene, which were chosen to partially cover portions of the shell domain and the P1 subdomain, respectively. Nucleotide sequences collected in Brazil and worldwide were compared chronologically and by molecular deduction involving several years with the same criteria and that was compatible with the sample collection period of the present study (between 1982 and 2011) or earlier. The viral divergence time was estimated, taking into account the date of a sample was isolated and the NoV nucleotide substitution rate for both genomic regions (i.e., C and D).

A maximum likelihood tree generated by the BEAST v. 1.7.5 package was applied, which also estimated the time to the most recent common ancestors (TMRCA) by means of a relaxed, exponentially uncorrelated lognormal molecular clock model [44]. The TMRCA was determined using the Coalescent Piecewise Bayesian Skyride Plot method [45] with 100 million replicates whose estimates were based on the GTR nucleotide substitution model. Trees were constructed using the ML method and using different models according to the genomic region analyzed: GTR+G (Pol/A), HKY+G (Pol/B), Tne+G (Cap/C), TIM2e+G (Cap/D) and GTR+G4 (Cap/P2) [46].

## Results

**Fig 1:**
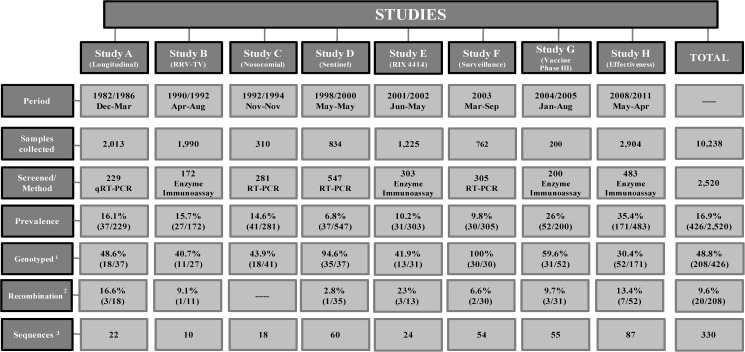
Flow chart of study samples demonstrating a global view of the study developed during a 30-year period of study (1982–2011) in Belém, Brazil. ^1^ Total of samples genotyped by at least one of the genomic regions analyzed. ^2^ Total of sequences used for recombination analysis. ^3^ Total of sequences used for phylogenetic analysis.

**Fig 2:**
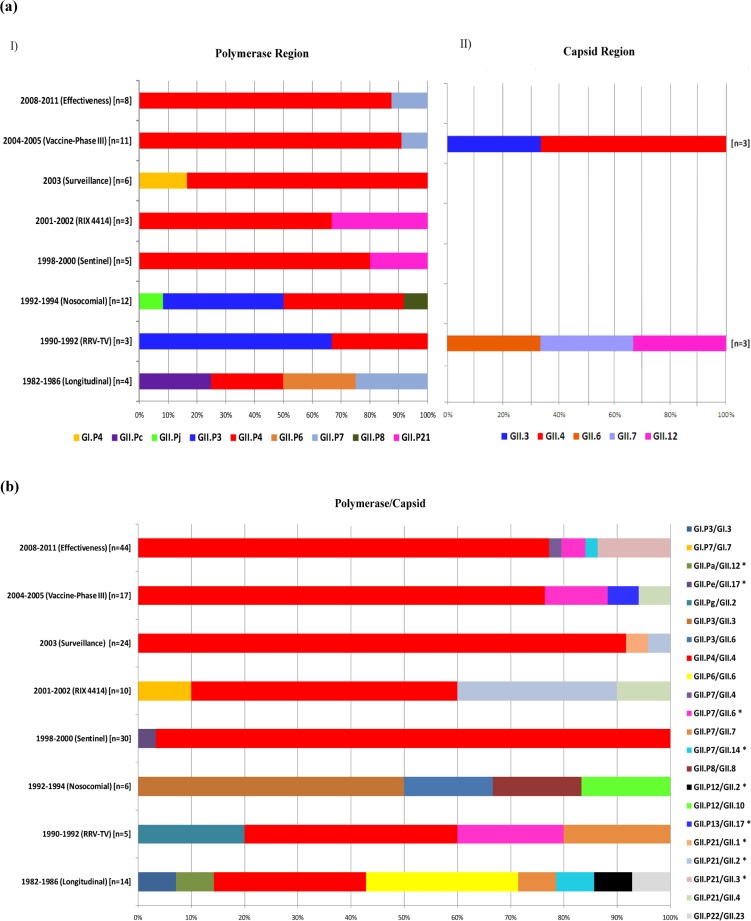
Norovirus genotypes detected in infected children during a 30-year period (1982–2011) in different studies conducted in Belém, Brazil. (a) (I) Samples genotyped only by a partial sequence of the polymerase gene (Regions A or B); (II) Samples genotyped only by a partial sequence of the VP1 gene (Regions C or D). (b) Binary genotyping targeted two regions, polymerase (A or B) and capsid (C or D). It is noteworthy that this study did not account for samples genotyped by only one nucleotide fragment (i.e., the polymerase or the capsid region). An asterisk represents samples confirmed as recombinants (more details are in Table 4).

A total of 2,520 stool samples from 2,396 cases involving young children and 124 controls without AGE were tested for the presence of NoV by a screening test available at the time, and an overall positivity of 16.9% (n = 426) was obtained, with 20.2% (25/124) observed in the asymptomatic group and 16.7% (401/2,396) in the symptomatic group (Fig 1). Table 2 shows the positivity rate obtained in each study from the period of sample collection. A sequence analysis of NoV specific RT-PCR products was obtained for 48.8% (208/426) of the positive samples, indicating a high genotype diversity during the study period in the polymerase gene (GI.P4, GII.Pa, GII.Pc, GII.Pe, GII.Pg, GII.Pj, GII.P3, GII.P4, GII.P6, GII.P7, GII.P8, GII.P12, GII.P13, GII.P14, GII.P21, GII.P22) and the VP1 gene (GI.3, GI.7, GII.1, GII.2, GII.3, GII.4, GII.6, GII.7, GII.8, GII.10, GII.12, GII.14, GII.17, GII.23). More details about the genotype distribution are in Table 3 and Fig 2A and 2B.

**Table 2:** Positivity rates of norovirus observed in each of the eight studies conducted between 1982 and 2011 in Belém, Brazil.

Period	Study		Description	Prevalence rate	References
1982–1986	A	Longitudinal	Community	16.1% (37/229)^*^	Siqueira et al. [14]
1990–1992	B	RRV-TV	Randomized clinical trials (Community)	15.7% (27/172)	Present study
1992–1994	C	Nosocomial	Cross-sectional (Hospital)	14.6% (41/281)	Present study
1998–2000	D	Sentinel	Cross-sectional (Hospital/Outpatient)	6.8% (37/547)^**^	Present study
2001–2002	E	RIX 4414	Randomized clinical trials (Community)	10.2% (31/303)	Present study
2003	F	Epidemiological surveillance	Cross-sectional (Hospital)	9.8% (30/305)	Aragão et al. [21]
2004–2005	G	Vaccine-Phase III	Randomized clinical trials (Community)	26% (52/200)	Present study
2008–2011	H	Effectiveness study	Case-control (Hospital)	35.4% (171/483)	Present study

^*^ A high prevalence rate was observed among the asymptomatic group (67.6%, 25/37) in comparison to the symptomatic group (32.4%, 12/37).

^**^A total of 271 samples were collected at the hospital (8.1% positivity, n = 22), and 276 were collected at a health unit (5.4% positivity, n = 15).

**Table 3:** Samples genotyped by maximum likelihood analysis of partial genome sequences from noroviruses detected in infected children in various collections during a 30-year period of study (1982–2011) in Belém, Brazil.

Gene/Region Fragment length (bp)	N of samples (Model test)	Genogroup I (n)	Genogroup II.4 (n)	Others GII (n)	
Pol/A 187, 236, 319, 327	135 (GTR+G)	GI.P4 (1) GI.P7 (1)	GII.P4? *(39) US_95/96 (17) Kaiso_2003 (31) Hunter_2004 (9) New Orleans_2009 (5)	GII.Pe (1) GII.Pg (1) GII.Pj (1) GII.P3 (11) GII.P7 (2)	GII.P8 (2) GII.P12 (1) GII.P13 (1) GII.P21 (12)
Pol/B 213	66 (HKY+G)	GI.P3 (1)	GII.P4? * (15) Bristol_1993 (1) Den Haag_2006b (19) New Orleans_2009 (9)	GII.Pa (1) GII.Pc (1) GII.P6 (5) GII.P7 (10)	GII.P12 (1) GII.P21 (2) GII.P22 (1)
Cap/C 390	65 (Tne+G)	—-	GII.4? * (5) US_95/96 (5) Asia_2003 (6) Kaiso_2003 (9) Hunter_2004 (2) Yerseke_2006a (1) Den Haag_2006b (5) New Orleans_2009 (7)	GII.1 (1) GII.2 (2) GII.3 (5) GII.6 (6) GII.7 (3)	GII.8 (1) GII.10 (1) GII.14 (2) GII.17 (3) GII.23 (1)
Cap/D 177, 253	86 (TIM2e+G)	GI.3 (1) GI.7 (1)	GII.4? * (11) US_95/96 (16) Asia_2003 (6) Kaiso_2003 (18) Den Haag_2006b (12) New Orleans_2009 (9)	GII.2 (2) GII.3 (5) GII.6 (4) GII.12 (1)	
Pol/A or B + Cap/C or D	150	GI.P3/GI.3 (1) GI.P7/GI.7 (1)	GII.P4/GII.4 (109)	GII.Pa/GII.12 (1) GII.Pe/GII.17 (1) GII.Pg/GII.2 (1) GII.P3/GII.3 (3) GII.P3/GII.6 (1) GII.P6/GII.6 (4) GII.P7/GII.4 (1) GII.P7/GII.6 (5) GII.P7/GII.7 (2) GII.P7/GII.14 (2)	GII.P8/GII.8 (1) GII.P12/GII.2 (1) GII.P12/GII.10(1) GII.P13/GII.17(1) GII.P21/GII.1 (1) GII.P21/GII.2 (4) GII.P21/GII.3(6) GII.P21/GII.4 (2) GII.P22/GII.23(1)

*“GII.P4?” and “GII.4?” correspond to GII.4 samples that could not be genotyped as variants using primers targeting regions A, B, C and D, from the polymerase and VP1 genes.

**Fig 3:**
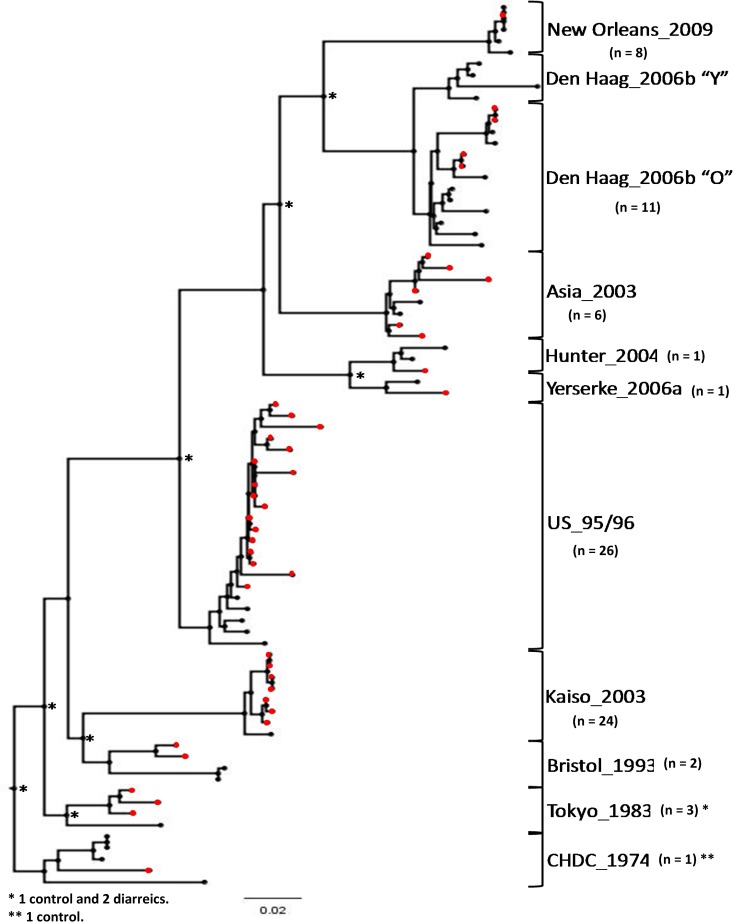
Maximum likelihood phylogenetic tree based on the P2 region of 83 partial genome sequences from noroviruses of different GII.4 variants detected in infected children during various collection periods over 30 years (1982–2011) in Belém, Brazil. Asterisks in the tree represent bootstrap values greater than 70% with 1000 replicates. Groupings of samples from the present study are in red. A dendrogram was constructed using model test GTR+G4. Variant reference strains used in the analyses were submitted to the GenBank database under the accession numbers CHDC_1970s (JX023286), Tokyo_1980s (AB684720), US_95/96 (DQ078829), Bristol_1993 (X86557), Kaiso_2003 (AB294779), Asia_2003 (AJ844476), Hunter_2004 (HM802544), Yerseke_2006a (EF126963), Den_Haag_2006b “O” (EF126965), Den_Haag_2006b “Y” (JX975571), and New_Orleans_2009 (GU445325).

**Fig 4:**
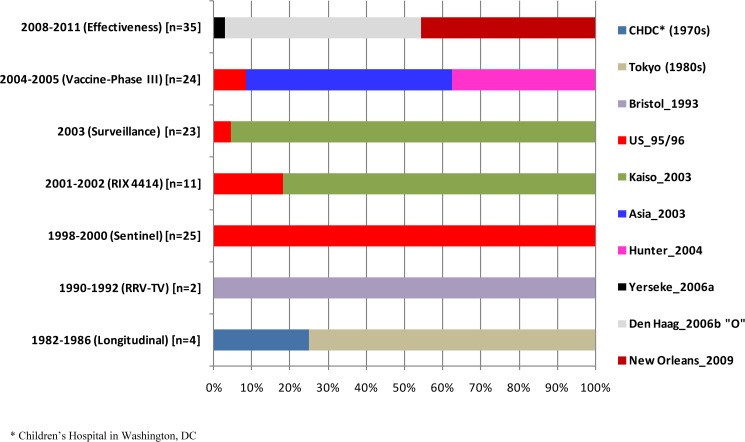
Temporal distribution of 124 samples classified as norovirus GII.4 variants according to the capsid region that were detected in infected children during a 30-year period (1982–2011) in Belém, Brazil. Of note, samples from Study C (nosocomial [1992–1994]) were not tested based on capsid region due to the depletion of these stools.

To determine the circulating GII.4 variants, the subdomain P2 region of 83 samples that genotyped as GII.P4/GII.4 by both ORFs (i.e., the polymerase and VP1 genes) was analyzed (Fig 3), and the circulation of 10 different variants over a 30 year period (1982–2011) was observed; the variants included CHDC (Children’s Hospital in Washington, DC [1970s]), Tokyo (1980s), Bristol_1993, US_95/96, Kaiso_2003, Asia_2003, Hunter_2004, Yerseke_2006a, Den Haag_2006b (subcluster “O”) and New Orleans_2009. The genotype distribution in the different study periods is shown in Fig 4.

Overall, 42 samples showed divergent genotypes between regions A or B (i.e., the polymerase gene) and C or D (i.e., the VP1 gene), suggesting that recombination had occurred. Of these, 20 (47.6%) were confirmed as being recombinant strains (Table 4): GII.Pa/GII.12, GII.Pe/GII.17, GII.P7/GII.6, GII.P7/GII.14, GII.P12/GII.2, GII.P13/GII.17, GII.P21/GII.1, GII.P21/GII.2, GII.P21/GII.3; intragenotype recombination included GII.P4 (US_95–96)/GII.4 (Kaiso_2003) and GII.P4 (Den Haag_2006b)/GII.4 (Yerseke_2006a). Different probable breakpoints within the viral genomes of the recombinant strains could be observed, and they ranged from 21 nt to 265 nt upstream and/or downstream of the junction region.

**Table 4:** Description of 20 recombinant norovirus strains circulating in the pediatric population of Belém, Brazil over a period of 30 years (1982–2011).

Study	Sample identification	Date of collection	Polymerase Region B	Prototype nt divergence	Capsid Region C	Prototype nt divergence	Breakpoin position at Junction *	Distance Overlap ORF1/2	RDP P-value	MaxChi P-value	BootScan P-value
(A) Longitudinal (1982–1986)	24333F17	01 Sep 1983	GII.P12	KJ196299 12.6%	GII.2	KC597138 4.9%	5038	64nt	1.5x10^-8^	5.5x10^-10^	9.7x10^-9^
	24145F25	09 Dec 1983	GII.P7	HM635119 5.6%	GII.14	KR904230 5.9%	5007	94nt	2.2x10^-3^	5.6x10^-7^	5.6x10^-3^
	24175F56	03 May 1985	GII.Pa	AF190817 3.4%	GII.12	AB045603 13.5%	5028	74nt	2.4x10^-5^	2.7x10^-2^	4.5x10^-6^
(B) RRV-TV (1990–1992)	COD066	08 Nov 1990	GII.P7	AF414409 6.9%	GII.6	JX989075 8.2%	5036	66nt	4.5x10^-3^	7.3x10^-5^	7.4x10^-3^
(D) Sentinela (1998–2000)	HST112	18 Jan 1999	GII.Pe	KU529177 2.3%	GII.17	KU561250 16.1%	5074	36nt	6.2x10^-8^	4.6x10^-11^	2.3x10^-11^
(E) RIX 4414 (2001–2002)	PID010	17 Feb 2002	GII.P21	AB542916 1.8%	GII.2	DQ456824 4.6%	5061	50nt	4.5x10^-11^	7x10^-11^	7.4x10^-10^
	PID158	15 Feb 2002	GII.P21	AB542916 1.5%	GII.2	X81879 6.1%	5066	35nt	2.3x10^-11^	2.3x10^-11^	2x10^-12^
	PID175	15 Feb 2002	GII.P21	AB542916 1.1%	GII.2	X81879 6%	5061	39nt	3.1x10^-11^	3x10^-9^	1.2x10^-10^
(F) Surveillance (2003)	VIG206	16 May 2003	GII.P21	AB542916 1.9%	GII.1	U07611 6.5%	5080	21nt	8.8x10^-9^	1.1x10^-9^	2.2x10^-9^
	VIG246	23 May 2003	GII.P4 (US95-96)	DQ078829 4.8%	GII.4 (Kaiso_2003)	AB186063 1.7%	4834	265nt	1.9x10^-3^	1.7x10^-5^	2x10^-3^
(G) Vaccine PhaseIII (2004–2005)	PID18252	20 May 2004	GII.P13	EU921354 1.2%	GII.17	LC037415 18.6%	5047	60nt	1.7x10^-4^	2.6x10^-4^	4.8x10^-5^
	PID18223	19 Aug 2004	GII.P7	AB258331 7.4%	GII.6	AB039778 6.8%	5027	74nt	7x10^-5^	1x10^-4^	7.8x10^-5^
	PID18548	03 Jun 2005	GII.P7	AB258331 6.6%	GII.6	AB039778 5.9%	5022	79nt	2x10^-5^	6.3x10^-5^	8.1x10^-6^
(H) Efectiveness (2008–2011)	2A0636	04 Nov 2008	GII.P21	AY682549 6.4%	GII.3	U02030 7.1%	5068	33nt	3.3x10^-8^	6.1x10^-9^	5.5x10^-7^
	2A0648	08 Nov 2008	GII.P21	AY682549 5.1%	GII.3	U02030 7.1%	5068	33nt	1.08x10^-8^	9.3x10^-12^	1.7x10^-7^
	2A0748	05 Dec 2008	GII.P21	AY682549 5%	GII.3	U02030 6.7%	5068	33nt	8x10^-9^	9.3x10^-12^	1.7x10^-7^
	2A1049	16 Feb 2009	GII.P4 (Den Haag 2006b)	EF126965 1.8%	GII.4 (Yerseke 2006a)	EF126963 1.1%	5002	48nt	4.7x10^-8^	4.3x10^-4^	4.7x10^-8^
	2A1118	03 Mar 2009	GII.P21	AY682549 5.1%	GII.3	U02030 7.1%	5068	33nt	2.6x10^-8^	9.3x10^-12^	7x10^-8^
	2A2620	19 Feb 2010	GII.P7	AF414409 6.6%	GII.6	JX989075 7.3%	5033	68nt	3.3x10^-4^	3.7x10^-3^	4.8x10^-4^
	2A2894	07 Apr 2010	GII.P7	AB258331 6.9%	GII.6	JX989075 8.2%	5024	77nt	1.1x10^-2^	1.3x10^-5^	8x10^-3^

* This analysis used the reference strain X459907 (complete genome).

**Fig 5:**
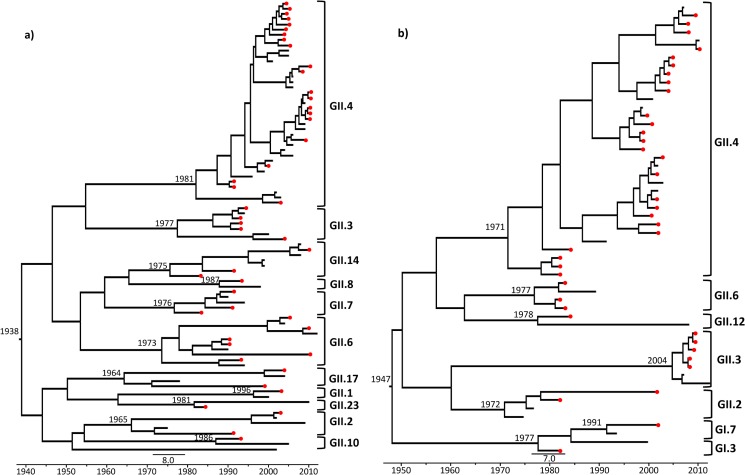
Maximum likelihood trees generated from relaxed and exponentially uncorrelated lognormal molecular clock model using the Coalescent Piecewise Bayesian Skyride Plot method with 100 million replicates (GTR nucleotide substitution model). This is a non-rooted tree representing the temporal distribution of the strains detected in infected children during various collection periods over 30 years (1982–2011) in Belém, Brazil. a) Represents samples classified by shell domain (Region C); b) Represents samples classified by P1 subdomain (Region D).

Evolutionary analyses to determine the rate of nucleotide subs./site/year were performed on the samples genotyped by regions C and D (Fig 5, S1 Table) and indicated a mean estimated rate of evolution of 1.02 x 10^−2^ and 9.05 x 10^−3^ subs./site/year, respectively.

Among all mutational sequences, those with the highest mutation rate in relation to genotype were the two belonging to genogroup I (GI.3: 1.24 x 10^−1^ and GI.7: 4.91 x 10^−2^ subs./site/year) in samples collected on June 08, 1983 and April 03, 2002, respectively. In genogroup II, the highest mutation rate was observed in a GII.2 sample dated February 15, 2002 (3.04 x 10^−2^ subs./site/year). Considering the genotypes for which at least three samples were evaluated by evolutionary analyses (GII.2, GII.3, GII.6 and GII.14), the Wilcoxon Rank-Sum Test (U) was applied to verify the differences among the mutation rates observed in GII.4 in relation to the others, and a statistically significant difference was not observed.

A Trimmed Mean of the TMRCA until the collection date of our samples was verified in order to avoid outliers that could bias the analyses. An average of 15.5 years of divergence for the GII.2 samples, 8 years for GII.14, 6.3 years for GII.4, 4 years for GII.6 and 2.7 years for GII.3 were observed. Four samples showed the longest divergence time of all evaluated: GII.2-COD401 (dated August 05, 1991), with 20 years since its TMRCA; GII.17-HST112 (dated January 18, 1999; TMRCA = 28 years); GII.6-2A2620 (dated February 19, 2010; TMRCA = 29 years); and GII.2-PID175 (dated February 15, 2002; TMRCA = 35 years). It is noteworthy that two GII.2 samples were verified to have the longest time of divergence since the MRCA, and one of them had the highest mutation rate of all genotypes from GII. More details about the evolutionary analyses involving the genotyped samples by regions C and D may be seen in Fig 5 and S1 Table.

## Discussion

NoVs have been progressively recognized as one of the more important pathogens of AGE worldwide since the 1990s, when molecular biology techniques became available for diagnosis [12,13,29]. However, few studies are available today that date back to samples collected before 1990, which makes it difficult to more comprehensively determine which strains circulated in the past and their relationship with contemporary strains.

### Prevalence rates

The overall positivity observed in this study (16.9%-426/2,520) corresponds to the results obtained in the 8 studies conducted during different collection periods in Belém, Brazil. However, it is important to note that each study had a different study design (i.e., cross-sectional, randomized clinical trial, case-control and longitudinal), which could explain the differences in the prevalence rates when the studies were analyzed individually (Table 2). Moreover, the various diagnostic techniques (i.e., qRT-PCR, commercial enzyme immunoassay and RT-PCR) used as the screening tests may have affected the prevalence rates, as indicated by differences in sensitivity and specificity, particularly in the laboratory tests. An additional point of consideration is the long-term storage or multiple freeze-thaw cycles to which the samples might have been subjected, which might have decreased the diagnostic accuracy of some of the tests. Therefore, this study did not aim to provide an accurate assessment of the incidence of NoV-associated illness but rather sought to approximate the circulation of the virus during the different periods studied.

Different prevalence rates were observed for the symptomatic (16.7%) and asymptomatic (20.2%) groups, and they were higher in the controls, possibly because shedding was prolonged in some cases. Preliminary data on the epidemiological aspects of the symptomatic and asymptomatic groups in a subset of these samples were previously published [14].

### Molecular epidemiology

A broad spectrum of genotypes was observed in this study; a total of 30 genotypes were determined, 16 by the polymerase gene and 14 by the VP1 gene (Fig 2A and 2B). There was a high prevalence of GII strains (n = 27) compared to the GI strains (only three: GI.P4 and GI.3, GI.7) throughout the study period. Although NoV genogroup GII is considered the most prevalent strain worldwide [46–49], in our study, this difference in the detection rates between GI and GII may be related to the designs of the eight studies. Note that most of the available published studies focus on hospitalized children or outpatients with severe diarrhea symptoms, similar to the present research, where most sample collection was primarily conducted in hospitals and health units, as well as in the community. Kitajima et al. [50] noted that genogroup I is commonly associated with a mild or asymptomatic infection that does not require hospitalization, while strains from genogroup II are more related to illness that requires clinical treatment.

Even though the relationship between virulence and infection by GI and GII remains speculative, our study reinforces the hypothesis that GII strains produce more serious illness, especially considering that GI had a similar prevalence as GII in a surface water surveillance study conducted in Belém that involved river, lake and sewer water [51]. The water surveillance study was conducted at the same time as Study H (2008–2011), indicating that GI infections that do not require medical care occur in the population. In Study A (1982–1986), a community-based longitudinal study, we collected samples from asymptomatic children and compared the frequencies of both genogroups. Full details on the epidemiological and clinical aspects of study A were published previously [14] and represented the first description of the GI.3 strain mentioned in the present paper. Details about GI.P4 were also previously published [21], but GI.7 was reported for the first time in diarrheic children in the present study.

GII.4 or GII.P4 was the only genotype that circulated throughout all sample collection periods, and it was the most prevalent from 1998 until 2011. Before then, this genotype was found with a frequency similar to that of the other circulating strains, such as GII.P6 and GII.P7 in Study A (1982–1986), GII.P3, GII.6 and GII.7 in Study B (1990–1992), and GII.P3 in Study C (1992–1994) (Fig 2A and 2B). In the current study, 10 different GII.4 variants circulating during the overall period and observed via a molecular analysis of the P2 region (Figs 3 and 4), of which, six strains are well established as pandemic variants (US_95/96, Asia_2003, Hunter_2004, Yerseke_2006a, Den Haag_2006b and New Orleans_2009) [35], the strains Bristol_1993 and Kaiso_2003 [52,53] had limited geographic circulation with no pandemic characteristics; and the two other strains (CHDC and Tokyo) were dated before the 1990s [54,55], for which time few studies about the molecular epidemiology of NoV are available, so it is difficult to accurately determinate the epidemic characteristics of these clusters.

Another genotype that had wide circulation in Belém between 1998 and 2011 was GII.P21. This strain was not detected prior to September 17, 1998, when it was possibly introduced into the population, but it was widely detected in Study E (2001–2002) and Study H (2008–2011), always in confirmed cases (Table 4) or in cases likely to have been caused by a recombinant strain (i.e., GII.P21/GII.2 in 2002, GII.P21/GII.1 in 2003 and GII.P21/GII.3 in 2008 and 2009). In 2013, Kroneman et al. [35] used the newly proposed 2xSD criteria, added GII.P21 to the established NoV genotypes, and observed that a virus found in India in 2006 (Hu/NoV/Ahm PC03/2006/India/EU019230 [56]) was well described as GII.b, but it was very similar to the new proposed genotype, so it was decided to rename the GII.b strains as GII.P21.

Some genotypes have been detected only once or twice in a period of 30 years, suggesting a limited circulation time (i.e., GII.Pa, GII.Pc, GII.Pe, GII.Pg, GII.Pj, GII.P8, GII.P12, GII.P13, GII.P14, GII.P22, GII.1, GII.8, GII.10, GII.12, GII.17 and GII.23). It is noteworthy that the greatest variety of strains was observed in Study A (1982–1986), most likely due to the longitudinal characteristics of the study. The longitudinal design allowed the children to be followed up for three years, with biweekly collections allowing the detection of NoV in subclinical cases and showing that a variety of strains were involved in the infections [14]. Because of the great genetic variability of this virus, it is worth emphasizing that to the best of our knowledge, some genotypes detected in the present study have not been previously described in Brazil (i.e., GII.Pj and GII.10).

### Recombination and evolutionary analyses

A broad spectrum of recombinant strains was detected during the 30 years of study in Belém (Table 4). The most prevalent was GII.P7/GII.6 (n = 5), which was observed in different years (i.e., 1990, 2004, 2005 and 2010), followed by GII.P21/GII.3 (n = 4) in 2008 and 2009, GII.P21/GII.2 (n = 3) in 2002 and GII.P7/GII.14 (n = 2) in 1983 and 2010. The other strains were detected only once during the entire study period. The most frequently found strain, GII.P7/GII.6, was first described in Northern Brazil in a sporadic case of diarrhea observed in patients who were in public health facilities from Manaus, in the Amazon Region [57], but our findings showed that this strain has been circulating in Belém since 1990 (i.e., 22 years before it was first described), at a distance of 1.293,37 km.

Few studies in Brazil targeted the detection of recombinant strains, but a paper on isolates from gastroenteritis outbreaks in Southern Brazil from 2004 to 2011 [58] reported some of the strains also found in the present study (i.e., GII.P7/GII.6, GII.Pe/GII.17, GII.P7/GII.14, GII.P13/GII.17 and GII.P21/GII.3), although our isolates had been detected in circulation years before in Northern Brazil. It is important to emphasize that the present study analyzed only confirmed recombinant cases (47.6%-20/42) (Table 4), but more laboratory and bioinformatics analyses of the other 22 samples are necessary to confirm recombination events and account for laboratory artifacts. This is a subject for a future publication. The presence of four possible intragenotype recombination events among the GII.4 variants is noteworthy and was confirmed for two events (Den Haag_2006b/Yerseke_2006a and US_95-96/Kaiso_2003), which have been more completely described in a recently published paper [59]. To the best of our knowledge, the recombinant strain GII.P21/GII.1 that was detected in the present study has not yet been described elsewhere.

The presence of five orphan clusters (GII.Pa, GII.Pc, GII.Pe, GII.Pg and GII.Pj), which were defined based on the partial polymerase genotyping system proposed by Bull et al. [60] for orphan polymerase sequences such as these, is notable, and such clusters have commonly been reported in association with recombination events. Note that some VP1 genotypes, e.g., GII.2 and GII.12, seem to be more prone to recombination than others and have been found in several clusters, some of which are orphan clusters [35]. Such an association was observed in our GII.Pg sample, as well as in another sample (24175F56) dated May 03, 1985, which genotyped as GII.12, was confirmed as a recombinant with the orphan polymerase GII.Pa strain.

The orphan cluster GII.d was also detected but was described herein as GII.P22 according to the classification proposed by the Norovirus Working Group [35], and this cluster was associated with a non-confirmed case of recombination with GII.23 by its VP1 sequence on March 13, 1984. The GII.Pe genotype was detected in a case of recombination with a GII.17 strain dated January 18, 1999. This strain was considered in the reclassification (NoroNet Nomenclature System) of the GII.4-Osaka_2007 strain and is currently intimately related with the latest Sydney_2012 variant strain, which has been found worldwide [61]. Another GII.17 strain dating from May 20, 2004, was also involved in a recombination event (GII.P13/GII.17). Preliminary analysis of the VP1 sequences showed no contemporary counterparts of this novel NoV variant, which was designated GII.17-2014 (KU687036; KP676383) and was primarily detected in environmental samples in Korea from 2004 to 2006 but also recently emerged in AGE outbreaks in China, Korea, Taiwan, Japan, Australia, France and the United States [62–65]. GII.17-2014 was recently described in hospitalized children from Belém [66], and more studies of this new strain are necessary to compare it to the old and new GII.17 genotypes and to verify any phylogenetic link between them.

Evolutionary analyses to determine the subs./site/year in our samples genotyped by regions C and D (Fig 5, S1 Table) had very similar overall mean estimated rates of evolution of 1.02 x 10^−2^ and 9.05 x 10^−3^ subs./site/year, respectively. Although region C comprises the N-terminal shell domain (inner viral capsid) and region D the C-terminal P1 subdomain (intermediary to the viral capsid), our findings suggest that the selective pressure on both regions of the genome is almost equal. Victoria et al. [67] also analyzed the evolutionary rates in region D of GII.4 samples from outbreaks and sporadic cases in hospitalized AGE patients from three public hospitals in the state of Rio de Janeiro, Brazil, and their results showed a rate 1.44 x 10^−2^ subs./site/year, which was higher than our results. However, their results and ours demonstrate an evolutionary rate that is comparatively higher than that observed for other RNA viruses, such as foot-and-mouth virus (complete genome: 8.2 x 10^−3^ subs./site/year) [68], echovirus 30 (VP1: 8.3 x 10^−3^ subs./site/year) [69], West Nile virus (ENV gene: 0.85 x 10^−3^ subs./site/year) [70], human immunodeficiency virus type 1 (gp160env: 2.4 x 10^−3^ subs./site/year) [71], human respiratory syncytial virus (G: 1.9 x 10^−3^ subs./site/year) [72] and hepatitis C virus (E2: 3.4 x 10^−3^ subs./site/year) [73].

A comparison genotypes from GI and GII showed a clear difference in the mutation rates, with that of GI (1.24 x 10^−1^ subs./site/year) being higher than that of GII (3.04 x 10^−2^ subs./site/year). Perhaps this high mutation rate of GI is a virulence factor of this genogroup, causing it to be clinically milder or asymptomatic and favoring its continued permanence in the population; however, this hypothesis is still speculative, and more studies are necessary to confirm it. Few studies involving the evolutionary rates of different genotypes are available, and most have targeted the GII.4 genotype [67,74].

In our samples, the subs./site/year observed for GII.4 was not significantly different compared to the other genotypes analyzed (GII.2, GII.3, GII.6 and GII.14), suggesting a similar pattern of viral evolution that is independent of genotype. Previous reports [67] have indicated the suitability of region D for evolutionary studies; however, when working with GII.4 species, the analysis of the P2 region of the VP1 gene is more appropriate due to its greater selective pressure. Moreover, the P2 region may reflect the subs./site/year of the entire VP1 gene, as has been previously asserted [74]. Unfortunately, the primers used in this study [32] to amplify the P2 region were designed exclusively for GII.4 strains, and it is not possible to characterize other clusters according to this genomic region. However, our GII.4 samples were defined by this region, and a more accurate analysis of evolutionary rates and the modeling of proteins of the variants described herein are in progress for future publication.

The trimmed mean of the time of divergence of the most recent common ancestors (TMRCA) for the collection date of our samples ranged from 2.7 to 15.5 years depending on the genotype. Four samples (two GII.2, one GII.6 and one GII.17) were the oldest since the MRCA to the sample collection date (ranging from 20 to 35 years). This suggests that these strains were stable in the population, even in cases with high mutation rates, as seen in strain GII.2-PID175, which dates from February 15, 2002 and showed the highest mutation rate of all GII genotypes in the current study.

These findings indicate that other mechanisms may have been involved in viral evolution over time, including recombination events, especially considering that the four samples that had the longest time of divergence until collection (GII.2-COD401; GII.2-PID175; GII.17-HST112; GII.6-2A2620) were recombinant strains (except for GII.2-COD401, which was highly likely to be a recombinant but was not confirmed as such).

More studies of evolutionary rates are necessary for the current samples and should involve the appropriate use of the complete VP1 gene or the P2 region to provide more robust data that may contribute substantially to our understanding of the initial transmission dynamics of NoV in the population of Belém and its viral evolution over time.

## Conclusion

This comprehensive study is the first study in Latin American with 30 years of sample collection and provides both new and historical insights into the molecular epidemiology of NoV infections in Brazilian children followed in the community, in outpatients and in hospitalized patients up to five years old with AGE. It also documents the broad genetic diversity of NoV and includes several recombinant strains (some novel and unusual), GII.4 pandemic variants and seasonal clusters. It provides a preliminary analysis of the evolutionary rate and some data that provide a better understanding of the complex dynamics of viral evolution and its variations, such as the higher mutation rate of GI in comparison to GII. A similar pattern of the evolution of GII.4 was also observed in relation to the other types (GII.2, GII.6 and GII.17) and was involved in the mechanisms of recombination with other strains. These findings may contribute to other studies that aim to develop a potential NoV vaccine or antiviral drug, which may depend on the knowledge reported herein about NoV genetic variation and molecular epidemiology over time worldwide. These are the first comprehensive analyses related to NoV circulation in the Northern Brazilian Amazon Region that encompass a 30-year period.

## Supporting information

S1 Table Norovirus evolutionary analyses demonstrating the nucleotide substitutions per site per year in samples genotyped by region C and D of the VP1 gene from samples collected in a 30-year period of study (1982–2011) in Belém, Brazil. (DOC)
