# Supplementary material for: Genotype diversity and molecular evolution of noroviruses: A 30-year (1982-2011) comprehensive study with children from Northern Brazil
Source: PLoS One. 2017 Jun 12;12(6):e0178909. doi: 10.1371/journal.pone.0178909 (PMC5467842; doi:10.1371/journal.pone.0178909)
Supplement: S1 Table — (DOC) [file pone.0178909.s001.doc]

|  | | **Region C** | | |  | **Region D** | | |
| --- | --- | --- | --- | --- | --- | --- | --- | --- |
| **Genogroup** | | **Sample code (collection date)** | **TMRCA^*^** | **Rate** |  | **Sample code (collection date)** | **TMRCA^*^** | **Rate** |
| **GI.3** | --- | | --- | --- |  | 24240F11 (08 Jun 1983) | 1977 | 1.24E-01 |
| **GI.7** | --- | | --- | --- |  | PID413 (03 Apr 2002) | 1991 | 4.91E-02 |
| **GII.1** | VIG206 (16 May 2003) | | 1996 | 4.83E-03 |  | --- | --- | --- |
| **GII.2** | COD401 (05 Aug 1991)  VIG136 (23 Apr 2003) | | 1971  2001 | 6.77E-03  4.28E-03 |  | PID175 (15 Feb 2002)  24333F17 (01 Sep 1983) | 1972  1972 | 3.04E-02  1.27E-02 |
| **GII.3** | 2 samples (1993 and 1994)  2 samples (1993)  PID18481 (21 May 2004) | | 1990  1990  1996 | 3E-03  4.56E-03  1.84E-03 |  | 2 samples (2008)  3 samples (2009) | 2007  2007 | 6.95E-03  2.21E-03 |
| **GII.4** | CHDC: 2 samples (1991)  Asia_2003: 9 samples (2004-2005)  Kaiso_2003: VIG057 (02 Apr 2003)  US_95/96: HST465 (27 Mar 2000)  Yerseke_2006a: 2A1049 (16 Feb 2009)  New Orleans_2009: 5 samples (2010)  Den Haag_2006b: 2 samples (2008 and 2009) | | 1990  1996  1998  1999  2005  2006  2007 | 2.23E-03  9.7E-03  2.26E-03  1.98E-03  2.97E-03  6.63E-03  2.62E-03 |  | CHDC: 3 samples (1983)/24175F51 (07 Feb 1985)  Kaiso_2003: 7 samples (2001-2003)  US_95/96: 5 samples (1999-2001)  Asia_2003: 5 samples (2004-2005)  Den Haag_2006b: 3 samples (2008-2009)  New Orleans_2009: 2A3013 (04 May 2010) | 1979/1978  1993  1994  2001  2004  2009 | 2,96E-03/5,67E-03  1.32E-03  4E-03  5.2E-03  8.88E-03  4.33E-03 |
| **GII.6** | 2A2620 (19 Feb 2010)  NSC053 (19 Jan 1993)  2 samples (1990)  PID18548 (03 Jun 2005)  2A2894 (07Apr 2010) | | 1981  1987  1988  2002  2008 | 3.54E-03  5.8E-03  3.25E-03  4.63E-03  2.21E-03 |  | 2 samples (1983 and 1984)  24018F32 (13 Jan 1984) | 1981  1982 | 3.7E-03  2.63E-03 |
| **GII.7** | 24195F27 (28 Dec 1983)  2 samples (1991) | | 1976  1984 | 1.21E-02  2.03E-03 |  | --- | --- | --- |
| **GII.8** | NSC296 (24 Dec 1993) | | 1987 | 9.75E-03 |  | --- | --- | --- |
| **GII.10** | NSC185 (17 Jun 1993) | | 1986 | 3,09E-03 |  | --- | --- | --- |
| **GII.12** | --- | | --- | --- |  | 24175F56 (03 May 1985) | 1978 | 2.10E-02 |
| **GII.14** | 24145F25 (09 Dec 1983)  COD099 (24 May 1991)  2A3231 (06 Jul 2010) | | 1975  1983  2007 | 6.07E-03  2.44E-03  2.72E-03 |  | --- | --- | --- |
| **GII.17** | HST112 (18 Jan 1999)  PID18252 (20 May 2004) | | 1971  1998 | 4.55E-03  2.51E-03 |  | --- | --- | --- |
| **GII.23** | 24333F31 (13 Mar 1984) | | 1981 | 1.66E-02 |  | --- | --- | --- |

**S1 Table** - Norovirus evolutionary analyses demonstrating the nucleotide substitutions per site per year in samples genotyped by region C and D of the VP1 gene from samples collected in a 30-year period of study (1982-2011) in Belém, Brazil.

*Time to most recent common ancestors.
